# Einstellungen gegenüber Menschen mit Demenz: Eine Bevölkerungsbefragung aus der Schweiz

**DOI:** 10.1007/s00391-021-01867-x

**Published:** 2021-03-17

**Authors:** Andreas Huber, Alexander Seifert

**Affiliations:** 1 Zentrum für Gerontologie, Universität Zürich, Pestalozzistraße 24, 8032 Zürich, Schweiz; 2 Universitärer Forschungsschwerpunkt (UFSP) „Dynamik Gesunden Alterns“, Universität Zürich, Zürich, Schweiz

**Keywords:** Demenzerkrankung, Alzheimer-Demenz, Einstellungen, Bevölkerungsbefragung, Schweiz, Dementia disease, Alzheimer’s disease, Attitudes, Population survey, Switzerland

## Abstract

**Hintergrund:**

Demenzerkrankungen, vorrangig die Alzheimer-Demenz, nehmen weltweit zu. Ein adäquater Umgang mit dieser Entwicklung erfordert den Einbezug der Bevölkerung in entsprechende Maßnahmen; ebenso erfordert er Kenntnisse über die Einstellungen der Bevölkerung im Hinblick auf die Erkrankung und die Menschen, die von dieser Erkrankung betroffen sind. Um die Einstellung der Schweizer Bevölkerung zu Menschen mit Alzheimer-Demenz oder anderen Demenzformen (MmAD) zu erheben und Faktoren herauszuarbeiten, die diese Einstellung beeinflussen, wurde eine Erhebung durchgeführt.

**Material und Methoden:**

Die schweizweite telefonische Befragung von 862 Personen ab 18 Jahren (M = 54,9 Jahre) fand in der Zeit von Juli bis September 2018 statt.

**Ergebnisse:**

Das Alter und die Einstellung zum Alter zeigten sich als signifikante Prädiktoren für die Einstellung zu MmAD. Die Unterteilung dieser Einstellung in eine kognitive und eine affektiv-konative Komponente erwies sich als hilfreich. Kontakt zur Zielgruppe, Informiertheit, erlebte Freuden und eine positive Einstellung zum Alter zeigen einen positiven Zusammenhang bei der affektiv-konativen Komponente, während sich Bildung, Geschlecht und Alter stärker auf die kognitive Komponente auswirken. Dennoch konnten die unabhängigen Variablen nur einen Teil der Varianz erklären.

**Schlussfolgerung:**

Trotz der insgesamt positiven Einstellung gegenüber MmAD in der Schweizer Bevölkerung ergeben sich aus den Ergebnissen dieser Studie Implikationen für die Praxis, die anzeigen, dass neben der Informiertheit auch die erlebten Kontakte zu MmAD die Einstellung zu diesen Personen positiv beeinflussen. Daraus ergibt sich, dass diese Kontakte gefördert werden sollten, um mögliche negative Bilder gegenüber MmAD zu revidieren.

## Abstract (en)

**Background:**

Dementia diseases, primarily Alzheimer’s disease, are on the rise worldwide. Adequate management of this development requires the involvement of the general population in appropriate measures; it also requires knowledge of the attitudes of the population with respect to the disease and the people it affects. A survey was thus conducted to discover the Swiss population’s attitude towards people with Alzheimer’s disease or related forms of dementia (ADRD) and identify the factors that influence this attitude.

**Material and methods:**

A nationwide standardized telephone survey of 862 people aged 18 years and older was conducted in German, French, and Italian between July and September 2018.

**Results:**

Age and attitude toward age were found to be significant predictors of attitudes towards people with ADRD. Subdividing this attitude into a cognitive and an affective conative component helped to more precisely assign the influences of independent variables. Regression models showed a positive effect on the affective conative component for contact with people with ADRD, being well informed, experienced pleasures, and a positive attitude toward age, while education, gender, and age had a positive impact on the cognitive component. Nevertheless, the independent variables could explain only part of the variance.

**Conclusion:**

Despite the overall positive attitude toward people with ADRD in the Swiss population, the results of this study have certain practical implications: in addition to being well informed, experience with people with ADRD has a positive influence on attitudes toward persons with this disease. Therefore, such contact should be encouraged to revise any negative perceptions of people with ADRD.

Das Verhalten der Bevölkerung gegenüber Menschen mit Alzheimer-Demenz oder anderen Demenzformen (MmAD) fußt auf deren Einstellungen zu diesem Krankheitsbild. Eine Schweizer Bevölkerungsbefragung untersuchte die Einstellung der breiten Bevölkerung zu MmAD. Es wurde hierbei der Frage nachgegangen, wie der persönliche Kontakt zu MmAD, das eigene Altersbild und das Interesse an sowie das Wissen über MmAD diese Einstellung beeinflussen.

## Hintergrund und Fragestellung

Aktuell leiden in der Schweiz 131.300 Menschen an Alzheimer-Demenz oder anderen Formen der Demenz („Alzheimer’s disease or related diseases“ [ADRD]) [1]. Die Alzheimer’s Disease International hat den Welt-Alzheimer-Bericht 2019 dem Thema „Einstellung zu ADRD“ gewidmet [2]. Dabei zeigte sich weltweit eine große Angst davor, einmal selbst eine ADRD zu entwickeln [2]. Die Einstellung gegenüber Menschen mit Alzheimer-Demenz oder anderen Demenzformen (MmAD) ist also tendenziell angstbehaftet.

Einstellungen können definiert werden als die Gesamtbewertung eines Einstellungsobjekts, die auf affektiven, kognitiven und verhaltensbezogenen Informationen beruht [3]. Einstellungen fußen also auf Überzeugungen, Gefühlen und eigenen früheren Verhaltensweisen. Das Verhalten einer Person gegenüber einem Einstellungsobjekt wird demnach von deren Einstellungen dazu beeinflusst [4]. Die Einstellung zu MmAD beeinflusst wiederum das Verhalten der Gesellschaft gegenüber diesen Menschen, also die Art, wie jenen Menschen begegnet wird, was ihnen zugetraut wird und was nicht oder was als hilfreich und finanzierungswürdig betrachtet wird [5]. Es kann angenommen werden, dass Einstellungen durch gezielte Maßnahmen in affektiver, kognitiver und verhaltensbezogener Hinsicht verändert werden können [6]. Um spezifisch die Einstellung und somit das Verhalten gegenüber MmAD verändern zu können, ist es notwendig herauszufinden, durch welche Faktoren die Einstellung zu MmAD beeinflusst wird. Verschiedene Studien haben sich bereits damit befasst. Cheston, Hancock und White stellten z. B. fest, dass das Wissen über ADRD sowie persönliche Erfahrungen die Einstellungen zu ADRD beeinflussen [7]. Diese Befunde zeigen sich auch in einer Schweizer Studie [8]. Darüber hinaus konnten verschiedene Studien Zusammenhänge zwischen der Einstellung zu MmAD und soziodemografischen Faktoren (z. B. Geschlecht [7, 9], Bildung [9], Alter [7] oder erlebten Freuden [8]) darlegen.

Diesbezügliche Befragungen wurden bereits in einigen Ländern entweder anhand von selbst erstellten Fragensammlungen [10] oder anhand von standardisierten Erhebungsinstrumenten [11] durchgeführt. Um eine gute Vergleichbarkeit zwischen den Studien verschiedener, aber auch innerhalb derselben Länder zu gewährleisten, ist es hilfreich, jeweils die gleichen Befragungsinstrumente zu verwenden. Die Dementia Attitude Scale (DAS) ist ein in den USA entwickeltes Messinstrument zur Erhebung der Einstellung der Gesamtbevölkerung gegenüber MmAD. In einem mehrstufigen Verfahren mittels Interviews sowie explorativer und konfirmatorischer Faktorenanalysen ergab sich eine Zweifaktorenlösung mit den übergeordneten Faktoren „cognitive domain“, welche die kognitive Komponente der Einstellung erfasst, und der „affective and behavioral domain“, welche die affektiv-konative Komponente der Einstellung abbildet [11]. Dass die DAS immer wichtiger wird, zeigen verschiedene Studien, die sich auf dieses Instrument gestützt [12], es in neuen Ländern verwendet [13] oder in andere Sprachen übersetzt haben [14].

Bevölkerungsbefragungen in der Schweiz bringen die Herausforderung mit sich, eine mehrsprachige Bevölkerung zu berücksichtigen. Peng, Moor und Schelling [15] haben daher die DAS ins Deutsche, Italienische und Französische übersetzt und validiert. Im Jahr 2012 wurde zum ersten Mal bei einer landesweiten Bevölkerungsbefragung in der Schweiz mit 8 ausgewählten Items der übersetzten DAS-Versionen gearbeitet [8]. Die 8 Items repräsentierten die 2 übergeordneten Faktoren, die bereits von O’Connor & McFadden [11] gefunden wurden und sich auch in der übersetzten Version manifestierten [15]. Die 2 Subskalen wurden als kognitive Komponente (im Folgenden: Kog.-Index) und affektiv-konative Komponente (im Folgenden: Aff.-Index) benannt.

### Ziele

Das Ziel dieser Studie war eine aktuelle Erhebung der Einstellung gegenüber MmAD in der Schweizer Gesamtbevölkerung. Aufgrund der Ergebnisse vergangener Forschung sollten Faktoren untersucht werden, welche die Einstellung zu MmAD beeinflussen, und es sollte daraus ein Vorhersagemodell erstellt werden, um Rückschlüsse für die Praxis ziehen zu können. Wir erwarten, dass (1) Kontakte, (2) Informiertheit und Interesse sowie (3) Einstellungen zum Alter die Einstellung zu MmAD beeinflussen, indem Personen mit einem persönlichen Kontakt zu MmAD, Personen, die sich sowohl informiert als auch interessiert gegenüber dem Thema Alzheimer zeigen, und Personen mit einer positiven Einstellung gegenüber dem Alter eher eine positive Einstellung zu MmAD aufweisen als Personen ohne Kontakt, Personen, die nicht informiert und interessiert sind, und Personen, die eine eher negative Einstellung zum Alter haben.

## Studiendesign und Untersuchungsmethoden

### Stichprobe

Grundgesamtheit der Befragung war die in der Schweiz wohnhafte Bevölkerung ab 18 Jahren. Als ökonomisch vertretbares Datenerhebungsverfahren mit einer guten Stichprobenausschöpfung wurde eine computergestützte telefonische Befragung (CATI) verwendet. Die Adressaten für die Befragung wurden im Auftrag des Bundesamts für Gesundheit (BAG) nach einem Stichprobenplan (hinsichtlich Alter, Geschlecht und Sprachregion) aus den offiziellen Registerdaten des Bundesamts für Statistik (BfS) gezogen. Die Information zur Befragung erfolgte mittels eines brieflichen Ankündigungsschreibens mit dem Briefkopf des Bundesamts für Gesundheit (BAG). Zwischen Mitte Juli und Mitte September 2018 wurden die angeschriebenen Personen in allen Sprachregionen der Schweiz telefonisch zu ihrem Wissen, ihren Einstellungen und Erfahrungen bezüglich MmAD befragt. Die realisierte Stichprobe beinhaltet 862 Personen im Alter zwischen 18 und 96 Jahren (M = 54,9; SD = 18,6). Wie Tab. 1 zu entnehmen ist, sind 56% der Befragten Frauen und 44% Männer. Beim Bildungsstand zeigen sich fast die gleichen prozentualen Anteile von Sekundarstufe II und Tertiärstufe. Der Vergleich mit den offiziellen Bevölkerungsverteilungen zeigt eine gewisse überproportionale Anzahl von befragten Personen ab 60 Jahren (Tab. 1).

**Table 1:** 

Stichprobe				Grundgesamtheit (Schweizer Bundesamt für Statistik)
Merkmal	Kategorien	Anzahl	Prozente	Prozente
Altersgruppen	18–29	105	12,2	17,8
	30–39	105	12,2	17,2
	40–49	115	13,3	17,5
	50–59	151	17,5	18,2
	60–69	160	18,6	13,2
	70–79	151	17,5	9,9
	80+	75	8,7	6,6
Geschlecht	Frauen	483	56,0	50,8
	Männer	379	44,0	49,2
Bildungsstand	Obligatorische Schule	80	9,4	12,2
	Sekundarstufe II	410	48,1	45,2
	Tertiärstufe I	183	21,5	42,6
	Tertiärstufe II	180	21,1	
Subjektive Gesundheit	Sehr schlecht	18	2,1	
	Eher schlecht	18	2,1	
	Teils, teils	101	11,8	
	Eher gut	389	45,4	
	Sehr gut	331	38,6	
Erlebte Freuden	Gar nichts	1	0,9	
	Wenig	26	3,0	
	Einiges	140	16,4	
	Viel	330	38,6	
	Sehr viel	357	41,8	
Kontakt zu Menschen mit Alzheimer	Ja	554	65,3	
	Nein	295	34,7	
Subjektive Informiertheit	Sehr schlecht informiert	39	4,6	
	Eher schlecht	159	18,8	
	Mittel	359	41,6	
	Eher gut	211	25,0	
	Sehr gut informiert	77	9,1	
Interesse am Thema ADRD	Überhaupt nicht interessiert	59	6,9	
	Eher nicht interessiert	276	32,4	
	Eher interessiert	392	46,0	
	Sehr interessiert	125	14,7	
Entwicklungsgewinne	M: 11,48, SD: 2,20	–		
Entwicklungsverluste	M: 9,58, SD: 2,37			

### Abhängige Variable

Die abhängige Variable dieser Studie war die Einstellung zu MmAD. Zur Erhebung wurden 8 Items aus der übersetzten DAS-Version [15] verwendet. Die 8 Items bilden – angelehnt an die vollständige übersetzte Version der DAS – die beiden Summenindizes Kog.-Index und Aff.-Index. Die Items und Summenskalen sind in Tab. 2 einzusehen. Obwohl die Items nach Alzheimer-Demenz fragten, wurden in die einleitende Fragestellung sämtliche Formen der Demenz miteinbezogen. Die Teilnehmenden wurden vor der Befragung darüber aufgeklärt. Für die Berechnung dieser Summenindizes wurden die Daten aller Personen, die eines oder mehrere der 4 Items eines Index mit „weiß nicht“ oder gar nicht beantwortet hatten, ausgeschlossen.

**Table 2:** 

Item	Englisch (DAS)	Deutsch	M/SD	Faktorladung Kog.-Index^a^	Faktorladung Aff.-Index^a^
Item 1	We can do a lot now to improve the lives of people with ADRD	Heutzutage können wir vieles tun, um das Leben von Menschen mit Alzheimer zu verbessern	4,01/0,906	0,493	0,061
Item 2	People with ADRD can be creative	Menschen mit Alzheimer können kreativ sein	3,72/1,160	0,742	0,051
Item 3	People with ADRD can feel when others are kind to them	Menschen mit Alzheimer spüren, wenn andere nett zu ihnen sind	4,22/0,970	0,601	0,123
Item 4	People with ADRD can enjoy life	Menschen mit Alzheimer können das Leben genießen	3,47/1,194	0,746	0,140
Kog.-Index Summenscore (Range: 4–20)	–	–	14,41/3,560	–	–
Item 5	I am afraid of people with ADRD	Ich habe Angst vor Menschen mit Alzheimer	1,40/0,833	0,015	0,653
Item 6	I am not very familiar with ADRD	Ich bin nicht sehr vertraut mit Alzheimer	3,08/1,377	0,020	0,488
Item 7	I would avoid an agitated person with ADRD	Ich würde einen aufgewühlten Menschen mit Alzheimer meiden	1,91/1,144	0,138	0,767
Item 8	I cannot imagine caring for someone with ADRD	Ich kann mir nicht vorstellen, mich um jemanden mit Alzheimer zu kümmern	2,40/1342	0,270	0,631
Aff.-Index Summenscore (Range: 4–20)	–	–	14,92/3,140	–	–

*M* Mittelwert, *SD* Standardabweichung

^a^Rotationsmethode Oblimin mit Kaiser-Normalisierung

### Unabhängige Variablen

Die unabhängigen Variablen dienten der Vorhersage der Einstellung gegenüber MmAD. Um herauszufinden, ob die Befragten Kontakt zu MmAD haben, wurde eine dichotome Variable (0: kein Kontakt, 1: Kontakt) verwendet. Daneben sollte untersucht werden, wie groß das Wissen über und das Interesse an ADRD ist. Dazu wurden 2 zusätzliche Variablen herangezogen: a) subjektive Informiertheit („Wie gut fühlen Sie sich über Alzheimer oder andere Formen von Demenz informiert?“; Skala 1 „sehr schlecht informiert“ bis 5 „sehr gut informiert“) und b) Interesse am Thema Alzheimer („Wie sehr interessieren Sie sich im Allgemeinen für Informationen über Alzheimer?“; Skala 1 „überhaupt nicht interessiert“ bis 4 „sehr interessiert“). Da ADRD oft in einen Zusammenhang mit einem hohen Alter gebracht wird [2], wurde auch die Einstellung zum Alter erhoben. Hierzu wurden die Subskalen „Entwicklungsgewinne“ (bestehend z. B. aus den Items „Ältere Menschen haben mehr innere Ruhe als jüngere“ und „Das Alter ist eine sehr schöne Lebensphase“) und „Entwicklungsverluste“ (z. B. Items „Ältere Menschen sind häufig deprimiert“ und „Im hohen Alter haben viele Menschen geistig abgebaut“) verwendet [16]. Als Kontrollvariablen wurden das Geschlecht, das Alter, die Bildung, die subjektive Gesundheit („Wie schätzen Sie Ihre Gesundheit zum heutigen Zeitpunkt insgesamt ein?“) und die Bewertung der Freude („Wie viel gibt es gegenwärtig in Ihrem Leben, das Ihnen Freude macht?“) in die Analysen integriert. In Tab. 1 sind die Ausprägungen aller in die Analysen einbezogenen Variablen und deren Verteilungen innerhalb der Stichprobe ersichtlich.

## Ergebnisse

### Deskriptive Ergebnisse

65,3% der befragten Personen gaben an, aktuelle oder frühere Kontakte zu MmAD zu haben (Tab. 1). Die Mehrheit der Personen fühlt sich „mittel“ (42%) oder eher gut informiert (25%) über ADRD. Das Interesse am Thema ADRD ist bei der Mehrheit eher vorhanden (46%); 32% gaben aber an, eher nicht interessiert zu sein. Die befragten Personen sahen im Alter mehr Entwicklungsgewinne (M = 11,48) als Entwicklungsverluste (M = 9,58).

### Faktorenanalyse

Die Struktur der 8 übersetzten DAS-Items zur Erhebung der Einstellung zu MmAD wurde mittels einer explorativen Faktorenanalyse untersucht. Sowohl der Bartlett-Test (Chi-Quadrat (120) = 431,235; *p* < 0,001) als auch der Kaiser-Meyer-Olkin-Test (KMO = 0,659) weisen darauf hin, dass sich die Variablen für eine Faktorenanalyse eignen. In Anlehnung an ähnliche Studien [8, 11] wurde eine Oblimin-Rotation mit Kaiser-Normalisierung durchgeführt. Nach dem Kaiser-Kriterium ergab sich eine Zwei-Faktoren-Lösung („Kog.-Index“ und „Aff.-Index“; Tab. 2), die 42,6% der Varianz erklärt. Um die interne Konsistenz der beiden Summenindizes zu bestimmen, wurde Cronbachs Alpha für die beiden Summenindizes berechnet. Die gefundenen Reliabilitätswerte (Cronbachs Alpha: Kog.-Index: 0,562; Aff.-Index: 0,459) sind zwar eher als schwach einzustufen, jedoch weisen beide Faktoren gute Abgrenzungen auf, sodass wir beide Faktoren zumindest als Summenindizes nutzen.

### Regressionsmodell zur Vorhersage der Einstellung zu MmAD

In einem ersten Schritt wurden mittels bivariater Regressionen die Zusammenhänge der unabhängigen Variablen auf beide abhängigen Variablen (Kog.-Index und Aff.-Index) untersucht. Von den unabhängigen Variablen nahmen bivariat das Geschlecht, die Bildung, die erlebte Freude und die Einstellung zum Alter als Entwicklungsgewinn statistisch signifikant Einfluss auf den kognitiven Einstellungsindex. Auf den affektiv-konativen Einstellungsindex nahmen bivariat die erlebte Freude, der Kontakt zu MmAD, die subjektive Informiertheit, das Interesse und die Einstellung zum Alter (Gewinne und Verluste) signifikant Einfluss. In einem weiteren Schritt wurden je 2 Regressionsmodelle für die beiden abhängigen Variablen gerechnet (Tab. 3). Für beide abhängigen Variablen wurde sowohl ein Modell erstellt, das lediglich die demografischen und personenbezogenen Variablen enthält, als auch ein Modell, das zusätzlich Komponenten in Bezug auf das Thema ADRD und die Einstellung zum Alter beinhaltet.

Das Gesamtmodell für den Kog.-Index (Modell 2) ist signifikant und kann 6,8% der Varianz erklären. Es ist abzulesen, dass Alter, Geschlecht, Bildung und die Einstellung zum Alter als Entwicklungsgewinn signifikante Prädiktoren sind. Frauen, jüngere Menschen sowie Menschen mit höherer Bildung und Menschen, die im Alter eher einen Entwicklungsgewinn sehen, zeigen höhere Werte auf den Kog.-Index als Männer, ältere Personen, Personen mit niedrigem Bildungsstand und Personen, die im Alter weniger Entwicklungsgewinne sehen.

**Table 3:** 

		Kog.-Index			Aff.-Index	
Prädiktoren	Bivariate Regressionen	Modell 1	Modell 2	Bivariate Regressionen	Modell 3	Modell 4
	*Beta*	*Beta*	*Beta*	*Beta*	*Beta*	*Beta*
Alter^a^	−0,072	−0,056	−0,097*	−0,031	−0,046	0,092*
Frau^b^	0,077*	0,080*	0,083*	−0,062	−0,062	−0,031
Bildung^c^	0,168***	0,158***	0,158***	−0,055	−0,052	−0,015
Subjektive Gesundheit^d^	0,065	0,018	0,001	−0,046	−0,021	0,002
Freude^e^	0,163***	0,116**	0,076	−0,134***	−0,125**	−0,081*
Kontakt zu Menschen mit ADRD (ja/nein)	0,065	–	0,033	−0,241***	–	−0,171***
Subjektive Informiertheit^f^	0,039	–	−0,003	−0,263***	–	−0,202***
Interesse an Alzheimer^g^	0,072	–	0,044	−0,170***	–	−0,072
Entwicklungsverluste^h^	−0,067	–	−0,025	0,124***	–	0,120**
Entwicklungsgewinne^h^	0,169***	–	0,158***	−0,124***	–	−0,086*
df1/p	–	5/<0,001	10/<0,001	–	5/<0,001	10/<0,001
Adjusted R^2^	–	0,048	0,068	–	0,020	0,133
N (gültige)	–	658	650	–	784	771

*Beta* Standardisiertes Beta

*p < 0,05; **p < 0,01; ***p < 0,001

^a^Alter in Jahren (18–96)

^b^Geschlecht, Dummy-kodiert (Frau: 1, Mann: 0)

^c^Bildung (1 „obligatorische“ – 4 „Universität/Hochschule“)

^d^Subjektive Selbstauskunft auf einer 5er-Skala (1 „sehr schlecht“ – 5 „sehr gut“)

^e^Subjektive Selbstauskunft auf einer 5er-Skala (1 „gar nichts“ – 5 „sehr viel“)

^f^Subjektive Selbstauskunft auf einer 5er-Skala (1 „sehr schlecht informiert“ – 5 „sehr gut informiert“)

^g^Subjektive Selbstauskunft auf einer 4er-Skala (1 „überhaupt nicht interessiert“ – 4 „sehr interessiert“)

^h^Summenwert über 4 Items auf je einer 4er-Skala (1 „trifft voll zu“ – 4 „trifft überhaupt nicht zu“)

Das Gesamtmodell der abhängigen Variable Aff.-Index (Modell 4) ist signifikant und erklärt einen Anteil der Varianz von 13,3%. Im Modell ist abzulesen, dass Alter, erlebte Freuden, Kontakt zu MmAD, subjektive Informiertheit und Alter als Entwicklungsverluste und -gewinne signifikante Prädiktoren sind. Jüngere Menschen, Personen, die viel Freude erleben, Personen mit Kontakt zu MmAD, Personen mit Wissen über AD, Personen, die im Alter viele Entwicklungsgewinne und weniger Entwicklungsverluste sehen, zeigen einen höheren Wert beim Aff.-Index.

## Diskussion

Mittels der übersetzten DAS-Versionen (Deutsch, Italienisch und Französisch) konnte eine Befragung der Schweizer Bevölkerung durchgeführt werden. Die Ergebnisse der Studie lassen darauf schließen, dass gegenüber MmAD tendenziell eine positive und freundliche Haltung eingenommen wird, dass ein gutes Wissen über ADRD vorhanden ist und ein grundsätzliches Interesse am Thema existiert. Es zeigte sich, dass der Kontakt zu MmAD die affektiv-konative Einstellung zu MmAD entscheidend beeinflusst; sie weist einen positiven Zusammenhang mit dem Item vorhandener Kontakt auf. Auch die Einstellung zum Alter stellt einen wichtigen Faktor in Bezug auf die Einstellung zu MmAD dar. Personen, die Entwicklungsgewinne im Alter für möglich halten, zeigen bei beiden Komponenten der Einstellung zu MmAD eine positivere Haltung; sprich, sie haben höhere Werte bei der abhängigen Variable Kog-Index und niedrigere Werte bei der abhängigen Variable Aff.-Index. Jedoch sind die erklärten Varianzen der multivariaten Analysen eher niedrig, womit stärker wirkende unabhängige Variablen – die in dieser Studie nicht berücksichtigt wurden – für die Einstellungsskalen noch offenbleiben.

Dennoch zeigen die Ergebnisse der Studie, dass sowohl die kognitive als auch die affektiv-konative Komponente durch verschiedene Faktoren beeinflusst werden. So erwiesen sich die Prädiktoren Alter, erlebte Freuden, Kontakt zu MmAD, subjektive Informiertheit, Interesse am Thema Alzheimer und die Einstellung zum Alter (Entwicklungsgewinn oder -verlust) als signifikant für die Einstellung zu MmAD. Es gab jedoch große Unterschiede hinsichtlich der Vorhersagekraft bei den unabhängigen Variablen. Während Kontakt zu MmAD, Informiertheit und erlebte Freuden nur die affektive Komponente vorhersagen können, können Bildung und Geschlecht nur die kognitive Komponente vorhersagen. Da beide Komponenten offenbar durch unterschiedliche Faktoren beeinflusst werden, ist deren Unterteilung also äußerst sinnvoll. Ältere Menschen zeigen bei beiden Komponenten eine negativere Einstellung als jüngere. Dies hat sich auch in anderen Studien gezeigt [17], die diesen Effekt auf das (Nicht‑)Vorhandensein von Ressourcen und auf Ressourcenveränderungen zurückführen.

Im Hinblick auf die Vorhersage der affektiv-konativen Einstellung zu MmAD ist der Kontakt zu diesen entscheidend. Menschen, die Kontakt zu MmAD haben, gaben weniger Ängste im Umgang mit MmAD an. Dies kann u. a. durch den „Mere-exposure“-Effekt [18] erklärt werden, nach dem sich die Einstellung durch die häufige Konfrontation mit einem Reiz – in diesem Fall MmAD – positiv verändert. Die Ergebnisse der vorliegenden Studie lassen vermuten, dass durch vermehrte Kontakte insbesondere irrationale Ängste abgebaut werden und so eine positivere Einstellung gegenüber MmAD entwickelt werden kann. Eine positive Beeinflussung der Einstellung zu MmAD könnte z. B. versucht werden zu generieren, indem das Thema ADRD in der Öffentlichkeit bekannter gemacht wird und der Kontakt zu MmAD (z. B. durch Kontaktmöglichkeiten im öffentlichen Räumen wie einem Museum [12]) gefördert wird. Auf diese Weise kann einer Stigmatisierung dieser Menschen weiter entgegengewirkt werden [2]. Projekte, durch die MmAD mehr in die Mitte der Gesellschaft rücken, können dazu führen, dass sich die Einstellung der Bevölkerung ihnen gegenüber verbessert. In Zukunft sollte aber auch mehr Wissen über ADRD vermittelt werden, um auch auf diese Weise zu einer positiveren Einstellung beizutragen. Das könnte ein wichtiger Schritt sein – und zwar hin zu der von der Alzheimer’s Disease International gewünschten demenzfreundlichen Gesellschaft [2].

*Limitationen*. Neben den Stärken der nationalen Befragung der MmAD-bezogenen Einstellungslandschaft in der Schweiz sollten auch deren Limitationen benannt werden. Es fällt auf, dass bei der Regression der kognitiven Komponente der Einstellung als abhängige Variable deutlich weniger Personen miteinbezogen werden konnten. Dies ist besonders auf die Einstellungsaussage zurückzuführen, mit der eruiert werden sollte, ob MmAD kreativ sein können. Viele Personen gaben an, diese Frage nicht beantworten zu können. Es ist anzunehmen, dass diese Frage die Studienteilnehmer tendenziell überfordert hat.

Der ökonomischen Durchführbarkeit einer telefonischen Befragungsstudie halber wurde nicht die gesamte übersetzte Version der DAS, sondern die Kurzversion verwendet. Die Reliabilitätswerte der beiden Summenindizes ergaben schwache Reliabilitätswerte, jedoch wurden ähnliche Werte bereits bei Moor et al. [8] festgestellt. Die Reduktion der übersetzten DAS-Version auf jeweils 4 Items/Komponente ist also ein Unterfangen, das zwar eine ökonomischere Durchführung für eine telefonische nationale Befragung ermöglicht, sich aber etwas auf die Reliabilität auswirkt.

Die erklärten Varianzen der multivariaten Analysen sind eher gering, womit viele erklärende Faktoren für die Einstellungsskalen noch offenbleiben; weitere Studien sollten diese offenen Faktoren noch besser herausarbeiten.

Bei der durchgeführten Befragung handelt es sich um eine Querschnittsuntersuchung; Veränderungen innerhalb einer Person können daher nicht abgebildet werden. Für die weitere Forschung wäre es daher wünschenswert, individuelle Daten im Längsschnitt zu erheben, anhand derer die Einstellungsveränderung in Abhängigkeit von verschiedenen kontextuellen, zeit- und lebensgeschichtlichen Einflussfaktoren beobachtet werden könnte.

## Fazit für die Praxis

- Die befragten Personen sind gegenüber Menschen mit Alzheimer-Demenz oder anderen Demenzformen (MmAD) tendenziell positiv eingestellt.
- Der Bevölkerung mehr Wissen zum Thema Demenz zu vermitteln und ihr Interesse dafür zu wecken, kann dabei helfen, deren Einstellung gegenüber MmAD positiv zu beeinflussen.
- Durch Integration von MmAD in die Mitte der Gesellschaft, z. B. durch die Vermittlung von entsprechenden Kontaktmöglichkeiten, kann die Einstellung der Bevölkerung gegenüber MmAD positiv mitbeeinflusst werden.
